# Sintered fluorapatite scaffolds as an autograft-like engineered bone graft

**DOI:** 10.1002/jbm.b.35374

**Published:** 2024-02

**Authors:** Clark Nielson, Jayant Agarwal, James Peter Beck, Jill Shea, Sujee Jeyapalina

**Affiliations:** 1 The Orthopaedic and Plastic Surgery Research Laboratory, George E. Wahlen Department of Veterans Affairs Medical Center, Salt Lake City, Utah, USA; 2 Department of Biomedical Engineering, University of Utah, Salt Lake City, Utah, USA; 3 Division of Plastic Surgery, Department of Surgery, University of Utah School of Medicine, Salt Lake City, Utah, USA; 4 Department of Surgery, University of Utah School of Medicine, Salt Lake City, Utah, USA; 5 Department of Orthopaedics, University of Utah School of Medicine, Salt Lake City, Utah, USA

**Keywords:** bone regeneration, engineered bone graft, fluorapatite, hydroxyapatite, stromal vascular fraction

## Abstract

Hydroxyapatite (HA)-based materials are widely used as bone substitutes due to their inherent biocompatibility, osteoconductivity, and bio-absorption properties. However, HA scaffolds lack compressive strength when compared to autograft bone. It has been shown that the fluoridated form of HA, fluorapatite (FA), can be sintered to obtain this desired strength as well as slower degradation properties. Also, FA surfaces have been previously shown to promote stem cell differentiation toward an osteogenic lineage. Thus, it was hypothesized that FA, with and without stromal vascular fraction (SVF), would guide bone healing to an equal or better extent than the clinical gold standard. The regenerative potentials of these scaffolds were tested in 32 Lewis rats in a femoral condylar defect model with untreated (negative), isograft (positive), and commercial HA as controls. Animals were survived for 12 weeks post-implantation. A semi-quantitative micro-CT analysis was developed to quantify the percent new bone formation within the defects. Our model showed significantly higher (*p* < .05) new bone depositions in all apatite groups compared to the autograft group. Overall, the FA group had the most significant new bone deposition, while the differences between HA, FA, and FA + SVF were insignificant (*p* > .05). Histological observations supported the micro-CT findings and highlighted the presence of healthy bone tissues without interposing capsules or intense immune responses for FA groups. Most importantly, the regenerating bone tissue within the FA + SVF scaffolds resembled the architecture of the surrounding trabecular bone, showing inter-trabecular spaces, while the FA group presented a denser cortical bone-like architecture. Also, a lower density of cells was observed near FA granules compared to HA surfaces, suggesting a reduced immune response. This first in vivo rat study supported the tested hypothesis, illustrating the utility of FA as a bone scaffold material.

## 1 | INTRODUCTION

Globally, it has been reported that over 2 million surgical procedures requiring bone graft materials are performed annually.^1,2^ This number is expected to surge due to an uptick in orthopedic injuries, osteopenic bone disease, and an aging population. Although autologous bone is considered the gold standard for bone augmentation or replacement, harvesting sites for these materials are limited in number, as is the volume of bone available in an individual skeleton. Harvesting autologous bone also requires extending the duration of surgery with attendant increased infection risk, donor site pain, and surgical costs.^3^ Allograft bone tissue may be used to avoid these limitations of autograft bone. Still, healing is less robust when compared to autografts owing to the required decellularization and sterilization processes, which intentionally remove immunogenic marrow cells but also lower bone-incorporation properties.^4^ Theoretically, engineered bone scaffolding materials could obviate these problems intrinsic to naturally obtained bone.

An ideal biomaterial for repairing critical-size bone defects has yet to be found. In terms of common bone biomaterials, natural polymers like collagen and chitosan degrade much more quickly than bone and lack the necessary mechanical strength to serve as bone substitutes.^5^ Synthetic polymers, such as polylactic acid (PLA), polyglycolic acid (PGA), and polycaprolactone (PCL), have controllable mechanical and degradation properties but do not integrate directly with bone.^5,6^ Calcium phosphates (Ca-P), comprising synthetic hydroxyapatite (HA) and tri-calcium phosphate (TCP), are inherently osteoconductive, can be broken down gradually and absorbed by the body (bioabsorbable) and replaced with endogenous tissues by the body’s natural physiological processes.^7,8^ However, their mechanical strength, bioactivity, and structural properties do not equal those of native bone tissue.^9^

Interestingly, the widely accepted “diamond concept”^10^ defines four features that a bone substitute must possess to match the regenerative capacity of autograft material; they are: (1) osteoconductivity, (2) mechanical stability/porous architecture, (3) osteogenic growth factors, and (4) autologous osteogenic cells that accelerate bone remodeling. While porous Ca-P bioceramics, like HA and β-TCP, exhibit osteoconductivity and can be fabricated with the porosity needed for neovascularization, they are brittle and unsuitable for load-bearing applications. Importantly, sintering—a high-temperature heat treatment process—has been shown to improve the mechanical properties of apatites and the response of osteogenic cells to these sintered materials in vitro.^11,12^ Another promising technique that increases the resemblance of synthetic apatites to the native bone matrix is to incorporate certain anionic and cationic components that are naturally present in biological apatites.^13^ The inclusion of specific ions, even at very small concentrations, can profoundly affect the stabilities, surface structures, and strengths of apatites,^14^ and it was realized that these changes might introduce desirable biological effects such as antimicrobial properties or anabolic activity.^15,16^ For instance, HA (Ca_10_(PO_4_)_6_(OH)_2_) can be fluoridated to make fluorapatite (FA; (Ca_10_(PO_4_)_6_F_2_). It has been shown that the elastic modulus, microporosity, surface structure/charge, and degradation behavior of HA can be modulated through fluoride substitution and sintering.^17–19^ Since apatite surfaces are osteoconductive—that is, allow osteogenic cells to adhere, migrate, proliferate, and deposit bone on their surfaces—there is now an emphasis in regenerative medicine toward delivering osteogenic cells on apatite scaffolds.^20,21^

Previously, several in vitro studies were conducted using different cell lineages (stem cells, pre-osteoblasts, osteoblasts), and it was determined that cell adhesion, proliferation, and differentiation occur on sintered FA surfaces.^18,22,23^ Although autologous osteoblasts can be incorporated into the structure of porous Ca-P scaffolds, allowing them to be delivered directly to the bone injury site, this methodology of including laboratory-grown cells in clinical applications is a long way from FDA approval. Thus, less FDA-restrictive stem cell types, such as adipose-derived stem cells (ADSCs) that are present within the stromal vascular fraction (SVF), have become of interest for bone regeneration applications and may be combined with growth factors. It has also been reported that these stem cells selectively differentiate toward an osteogenic lineage on HA surfaces.^20,21^

The SVF is an excellent source of ADSCs, and it is reported to contain a heterogeneous cell population while consisting of ~20%–40% ADSCs, depending upon the isolation technique.^24^ These cells can be driven toward an osteogenic lineage by specific biological signals.^25^ In our earlier study, the data suggested that the differentiation of ADSCs to an osteoblast lineage occurred much faster on sintered FA surfaces than on HA, even without any additional growth factors.^21^ We have also previously shown that sintering temperatures strongly influence interactions between cells and apatite surfaces.^17,23^ In summary, our previous work shows that FA promotes cell adhesion, growth, and differentiation of stem cells more readily than HA in vitro.^21^ The data from these studies collectively indicate that the combination of autologous SVFs and FA scaffolds may produce an engineered-bone construct that is “autograft-like.” Thus, by adding the SVF to the porous, sintered FA scaffolds, all four features of the previously mentioned diamond concept may be achieved, which concept needs to be tested in a translational animal model.

This study was therefore designed to test the hypothesis that sintered FA scaffolds, with or without SVF, could regenerate bone tissue within the defects to an equal or greater extent than autograft materials or currently clinically utilized HA predicate bone scaffolds. To test this hypothesis, FA scaffolds were fabricated in-house, sintered, characterized, and combined with SVF to obtain autograft-like scaffolds. These scaffolds were then tested in a surgically created rat femoral condylar defect for 12 weeks.

## 2. | MATERIALS AND METHODS

### 2.1 | Materials

Unless otherwise stated, all chemicals were of analytical grade and purchased from Sigma Aldrich (St. Louis, MO).

### 2.2 | Synthesis and characterization

FA powders were synthesized using a previously described technique.^26^ Briefly, 750 mL of 1.2 M Ca(NO_3_)_2_ and 750 mL of a mixture containing equal parts of 0.54 M NaF + 0.72 M Na_2_H(PO_4_) were dispensed at a rate of 2.7 mL/min into a flask that contained 10-liters of boiling deionized (DI) water. The pH was maintained at ~9.16 using the auto-titrator containing 2 M NaOH. The precipitated FA powder was filtered, rinsed with DI water, and then dried and characterized in terms of fluoride, phosphate, and calcium concentrations, as per our prior publication.^17^ Fourier-transformed infrared (FTIR) spectroscopy (Nicolet iS50, ThermoFisher, Waltham, MA) and X-ray diffraction (XRD, Bruker D2 Phaser, Billerica, Massachusetts) were used to detect any impurities or secondary phase or unreacted reactants post-sintering (0–1150°C, 5°C/min, 2 h isothermal hold) using a high-temperature furnace (MODEL: Sentro Tech, Strongsville, OH, USA). Using a previously described protocol, the concentration of fluoride in acid-dissolved samples of sintered FA was determined using a fluoride ion-selective electrode (Hach Sension + 9655C, Loveland, CO).^17^

### 2.3 | Fabrication and characterization of porous FA/HA scaffolds

The well-known “lost foam” technique with modification was employed to create porous FA and HA scaffolds.^27^ HA scaffolds were produced using commercial HA powder (Hydroxyapatite Bio-Gel^®^ HTP Gel, BIO-RAD, Hercules, CA). For this, reticulated 30 pores-perinch polyurethane foam (Foam Mart, Burbank, CA) was cut into 2 × 1 × 1 cm^3^ rectangles and then immersed in colloidal FA or HA suspensions (50 wt% powder) stabilized with a dispersant (Darvan C-N, Vanderbilt Minerals, Gouverneur, NY), a surfactant (simethicone), and a binder (sodium carboxymethyl cellulose [CMC]). The excess slurry was removed using compressed air. The scaffolds were carefully dried and then heated in a furnace at 2°C/min to 300°C (2-h isothermal hold) and then heated at 2°C/min to 600°C (4 h isothermal hold) to vaporize the organic foam. After which, they were sintered at 1150°C with a 2 h isothermal hold. Since the resultant scaffolds were larger than the bone defects, they were crushed into small pieces (270 ± 210 μm) before sterilization and implantation.

### 2.4 | Animal model study design

Thirty-two male, inbred Lewis rats (004, Charles River, Wilmington, MA) were used. The animals were quarantined for 2 weeks prior to the procedure and had an average weight ~250 g. The number of samples/group needed to achieve 80% power to detect differences between autograft and apatite groups was determined using a power calculation. A total of 32 rats were used, but two were removed from the study. The remaining 30 rats were randomly divided into five groups of six rats (*n* = 6/group). The groups were defined as follows: Group 1, isograft (positive control); Group 2, no grafting material used (negative control); Group 3, HA scaffold (clinical standard); Group 4, FA scaffold; Group 5, FA scaffold combined with stromal vascular fraction (FA + SVF). Since the inbred animals are genetically similar, tibiae were harvested from Group 4 (FA scaffold) and used as donor tissue at necropsy to create the isograft material for Group 1. While isografts are technically not true autografts, for this small animal study, they were considered equal to autograft bone because of the limiting paucity of autologous donor sites. Therefore, the terms “isograft” and “autograft” were used interchangeably in this manuscript. Bone fragments were minced using a disposable clinically used bone mill (Bone mill V2.0, Merten, Germany) and then implanted into the defects of Group 1 (isograft). The abdominal adipose tissue of Group 2 (no grafting) was aseptically harvested at their respective necropsies, processed (see methods below), and used as the donor tissue for harvesting SVF for the Group 5 animals.

### 2.5 | Stromal vascular fraction extraction

Collected adipose tissue from donor rats (Group 2) was weighed, rinsed with lactated Ringer solution, and suspended in collagenase solution (0.15% Type 1 and 0.1% Type 2) for 30 min, centrifuged, and then SVF collected, following a previously clinically utilized protocol.^28^ The concentration of viable cells was obtained by collecting a small sample of cells, mixing this with Sphero™ AccuCount particles (Sperotech, Lakeforest, IL) and DAPI, and then analyzing the cell population using a BD FACSCanto™ fluorescent activated cell sorting (FACS, BD Biosciences, Franklin Lakes, NJ). Then, approximately 8 × 10^5^ viable cells were suspended in 50 μL Dulbecco’s Modified Eagle Media (DMEM, ThermoFisher, Waltham, MA) and mixed with 5 mg of FA scaffolds for each animal. The scaffolds were incubated for approximately 1 h at 37°C to allow the SVF cells to adhere, washed with PBS, and then implanted in a femoral defect.

### 2.6 | Surgical procedure

**FIGURE 1:**
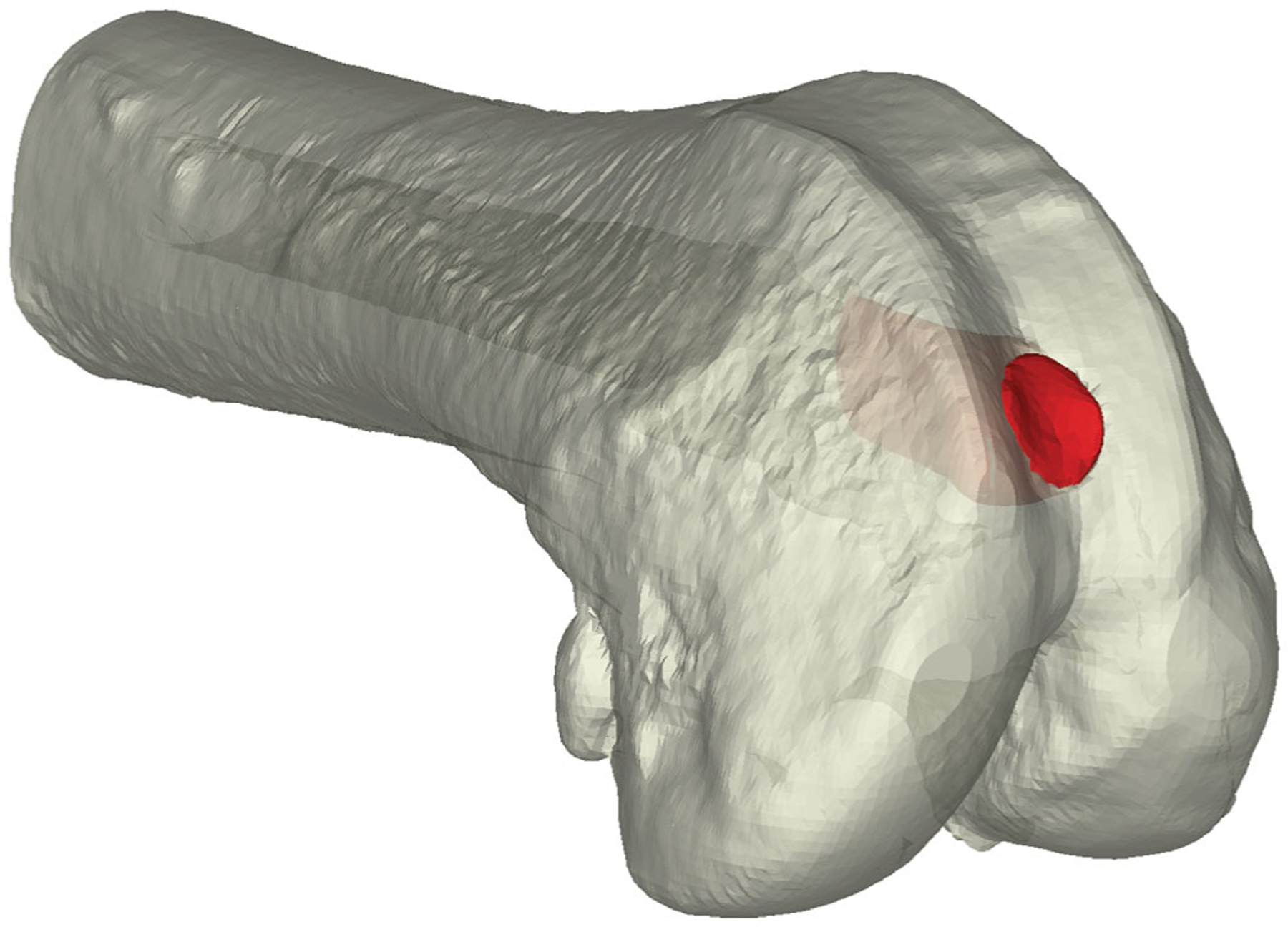
A schematical illustration of the defect (red).

Animals were prepared for aseptic surgery using standard procedures outlined in the locally approved IACUC (Salt Lake City VA # A20–01) protocol. Briefly, during the surgery, animals were anesthetized and maintained under anesthesia using isoflurane. The hair around the right knee was shaved, the skin prepared for surgery with Chlora-Prep^®^, and then the animals were positioned in the dorsal recumbent position. A longitudinal incision was made lateral to the patella to expose the femoral condyle and intercondylar notch. A bone defect (1.2 mm in diameter × 6 mm in depth, Figure 1) was then created from the center of the articulating surface and through the growth plate, parallel to the long axis of the bone, using a drill with a frequent saline flush. The defects were then treated according to the group assignment. Isograft tissues or small pieces of the scaffolding were tamped into the defect before closing the incision line with resorbable sutures (Vicryl absorbable sutures, Ethicon Inc., Cincinnati, OH). As the animal recovered, one subcutaneous (SC) injection of Rimadyl (Carprofen; 5 mg/kg) was given as postoperative analgesia. The rats were also given carprofen wafers (5 mg/100 g) once a day for up to 7 days post-surgery. The animals were housed in pairs and provided with standard enrichment. Technical support staff monitored the animals daily for signs of distress, such as limping, limited food/water consumption, or decreased activity levels. Those persons performing the histological and micro-CT analyses were not blinded to the groups.

### 2.7 | Micro-CT imaging

At post-surgery (*t* = 0) and necropsy (*t* = 12 weeks), animals were imaged with micro-CT (Quantum FX, Billerica, MA). The acquired images were processed using Mimics software (version 22.0, Materialize, Leuven, Belgium) for quantitative analysis.

### 2.8 | Semi-quantitative microCT analysis

A volume of interest (VOI) was created that centered along the longitudinal axis of the defect. The dimensions of the VOI were maximized to avoid the inclusion of dense cortical bone or bone at the growth plate region to ensure a more homogeneous distribution of bone structure within the VOI for the calculations.^29^ The width and length of the VOI varied between 1.0–1.2 and 0.8–1.9 mm, respectively. To measure bone volumes in the defects at 12 weeks, a trained operator created individual thresholds that differentiated soft, collagenous tissue from the mineralized bone within the VOI, using characteristic bone peaks in the counts versus Hounsfield units (HU) histogram. Then the masked VOIs were compared to histology slices to confirm the thresholds visually. Due to the resolution of the scans (voxel size = 40 μm^3^), small intra-trabecular spaces near dense trabecular bone or scaffolding were not captured by the soft tissue thresholding techniques; these volumes being added to the soft tissue mask manually using a higher threshold. Thresholds were determined while viewing slices of the VOI with contrast adjustments per set standardization. Additionally, thresholds were created to distinguish the scaffolding from mineralized bone tissue based on the histogram’s characteristic peaks (2250–4500 HU). Bone volume fraction (BVF), the volume of mineralized bone tissue per unit volume of sample, was calculated on a voxel basis.^30^ For the autograft group, the percentage of bone filling (%) within the VOI was calculated as the volume of bone (Vb) divided by the total volume of the VOI (Vt). For the FA, FA + SVF, and HA groups, scaffold volumes were subtracted from Vt to obtain total available volume of spaces for the bone tissue to in grow (V), and bone filling (%) was taken as Vb/V.

### 2.9 | Histology

At necropsy, harvested bone tissues were fixed with 10% neutral formaldehyde, dehydrated with ascending grades of alcohol, and embedded in polymethyl methacrylate (PMMA). Two-millimeter sections containing the defects were obtained using a linear saw (Isomet 4000, Buehler, Labe Bluff, IL, USA). Optically thin sections were prepared for staining by grinding and polishing (EcoMet^®^ 250 Grinder-Polisher; Buehler, Lake Bluff, IL). These sections were stained with bone stain (Sanderson’s RBS, DHM, Loxley, AL) for 10 min at 60°C and counter-stained with acidified eosin. The secondary outcome measure was newly formed bone on the surface of the implanted materials and the degree of integration with the surrounding tissue. The sections were then imaged using a transmission light and a 3D microscope. (VHX 6000, Keyence, Osaka, Japan).

### 2.10 | Statistical analysis

All data are reported as mean ± standard deviation unless otherwise stated. As determined using micro-CT analysis, group differences in percent bone volume within the defect were statistically compared to autografts and to defect-only groups using multiple student *t*-tests. An alpha of .05 was used as the cutoff for significance. Data available upon request.

## 3. | RESULTS

### 3.1 | Characterization

Characterization was performed in the same manner as reported in our previous study,^17^ and the important findings are summarized in Table 1. These results collectively indicated that post-sintering, the Ca/P ratio of our synthesized material was 1.42. The sintered scaffolds exhibited a fluoride content of 1.39 mol/kg, as determined by the fluoride electrode. This value was approximately 70% of the theoretically anticipated amount (1.98 mol/kg), assuming complete replacement of both hydroxyl groups in HA by fluorides. In addition to the expected FTIR bands for P-O, and O-P-O, bands associated with HPO_4_^2–^, CO_3_^2–^, NO_3_^–^, and OH^–^ were also seen in the FTIR spectrum of the FA powders (Supplementary Figure S1). Also, a significant band near 744 cm-1 developed post-sintering. The main phase was identified as FA from XRD analysis, and no other distinguishable secondary phases were detected (Supplementary Figure S2).

### 3.2 | In vivo model

A total of 32 Lewis rats was used in this study. Per IACUC protocol, two animals were euthanized early and removed from the study due to intra-operative complications (broken femur). Post-surgery, the remaining 30 rats in the study survived. They ambulated efficiently without showing any gait abnormalities or distress. The rats were housed communally, and no significant changes in their behavior, including lameness to the right hind limbs, were observed over the course of this 12-week study.

### 3.3 | Micro-CT imaging

**FIGURE 2:**
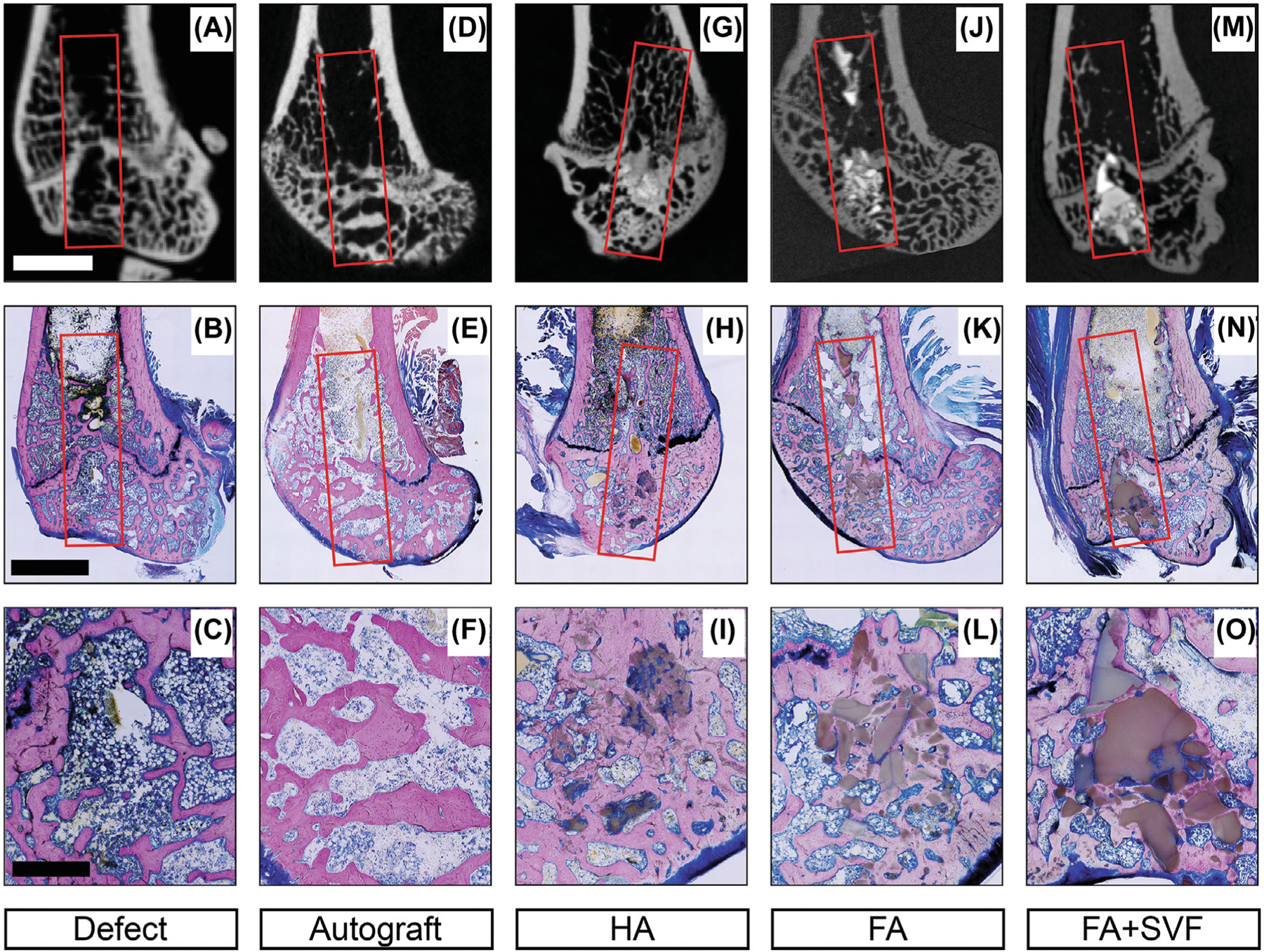
Representative microcomputed tomography (micro-CT) and histological images that have been aligned from each group. The top row shows micro-CT images taken at necropsy, while the bottom two rows display photomacrographs (middle) and photomicrographs (bottom) of rapid-bone stain and acidified eosin highlighting bone tissue in pink, scaffold in gray, and soft tissues/cell nuclei in blue. The scale bar represents 1 mm.

Representative micro-CT images taken at necropsy are given in Figure 2 (top row). The defect-only group clearly showed that, within the 12 weeks, the defects were not filled/bridged with bone. Few trabeculae were observed to span the periphery of the defect; for example, the mineralized bone tissues were only present at the periphery and not within the center of the surgically created defects (Figure 2A–C). In comparison, it appeared that the isografts became incorporated within the surrounding bone structure (i.e., trabecular bone Figure 2D–F), while the HA, FA, and FA + SVF groups showed new bone formation within the scaffolds (Figure 2G–O).

### 3.4 | Semi-quantitative analysis

**FIGURE 3:**
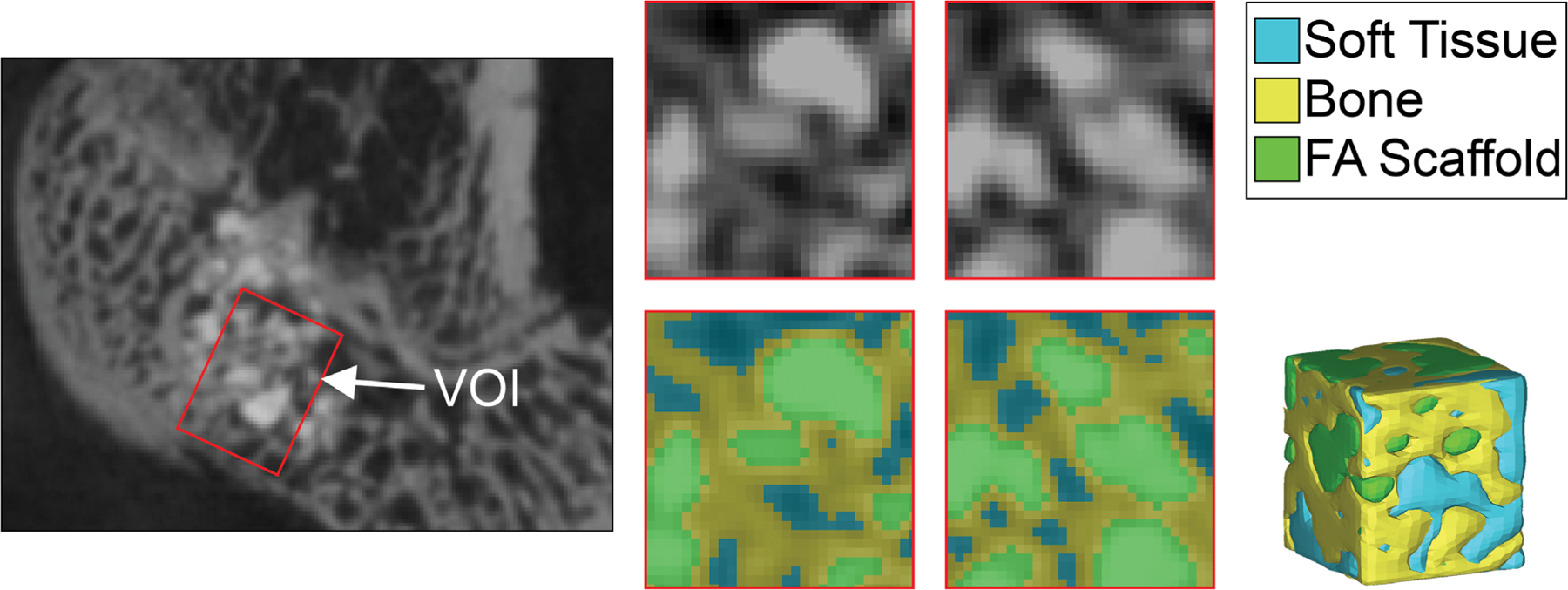
Microcomputed tomography (micro-CT) images of a femur from the fluorapatite + stromal vascular fraction (FA + SVF group, left) after 12 weeks. The volume of interest (VOI) is indicated by a red box. Slices of the VOI (middle, outlined in red) demonstrate the thresholding technique. The calculation of bone volume within the VOI involved assessing the volumes of soft tissue, bone, and scaffold. A 3D rendering of the VOI is presented in the bottom right. The scale bar corresponds to approximately 2 mm.

**FIGURE 4:**
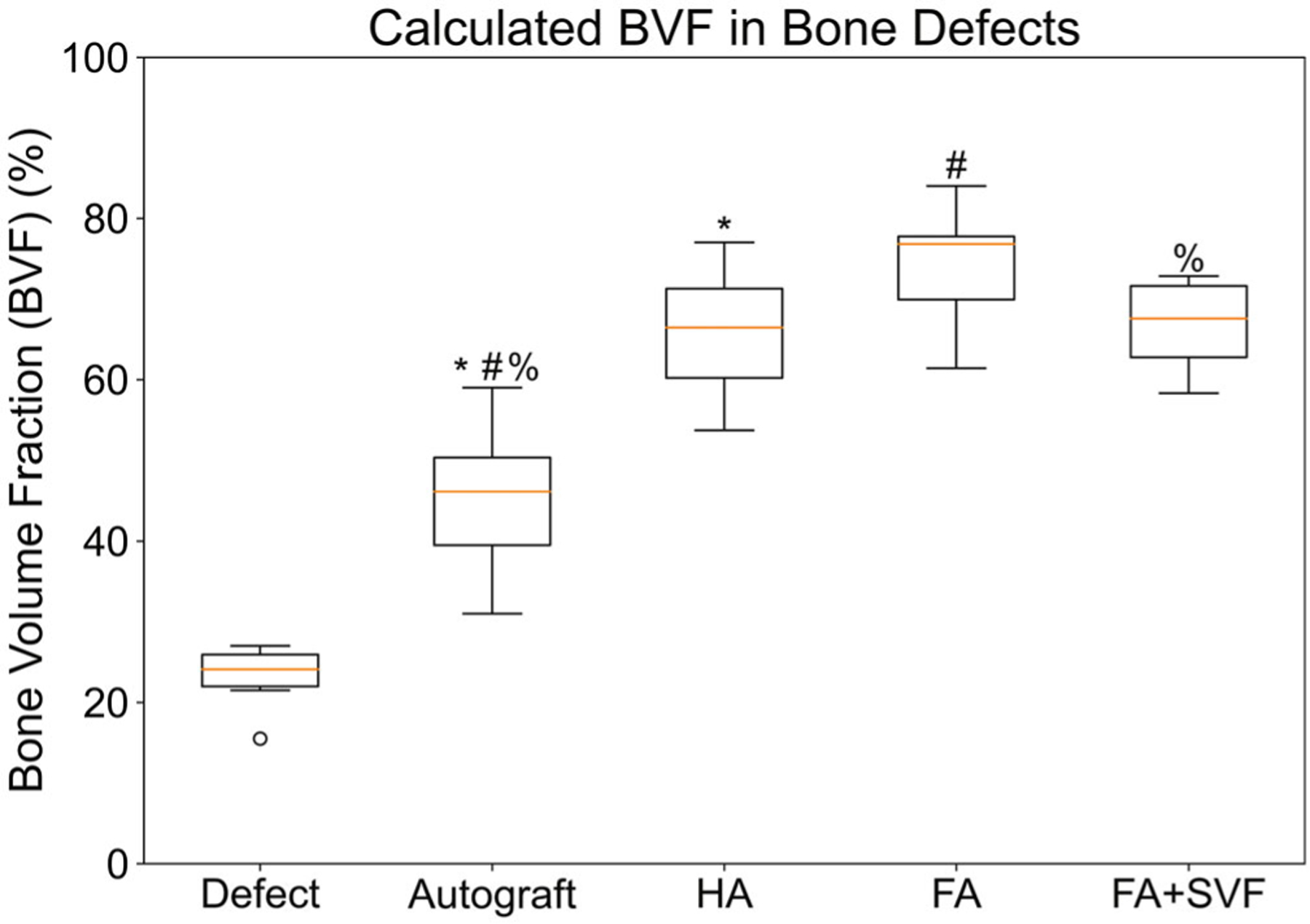
Box plot depicting the average bone volume fraction (BVF) (represented by the orange line) for each group using our semiquantitative method. The black box represents the interquartile range, with the top and bottom indicating the 3rd and 1st quartiles, respectively. The capped black lines depict the maximum and minimum values. Outliers are represented by circles. The fluorapatite (FA) group exhibited the highest average BVF. Significant differences were observed between FA, hydroxyapatite (HA), and FA + stromal vascular fraction (FA + SVF) groups compared to autograft (indicated by *, #, and %, respectively; p < .05).

Figure 3 depicts the methodology used for quantifying BVF. Within the VOIs, HU thresholds representative of bone, soft tissues, and scaffolds were applied to create volumes for visualization. Figure 4 presents the average calculated BVF within the VOI. These data clearly showed that defects treated with FA, HA, and FA + SVF had greater mean BVF than defect-only (23.5 ± 3.4%) (Figure 4). Pairwise t-tests revealed that the differences in BVF between HA (68.0%, ± 8.7%), FA (76.3%, ± 5.5%), and FA + SVF (66.8%, ±5%) compared to autograft (46.4% ± 10%) were statistically significant ( *p* < .05). Among the HA, FA, and FA + SVF groups, the FA group had the highest mean value, but no significant differences were found when HA versus FA, HA versus FA + SVF, and FA versus FA + SVF were compared.

### 3.5 | Histology

**FIGURE 5:**
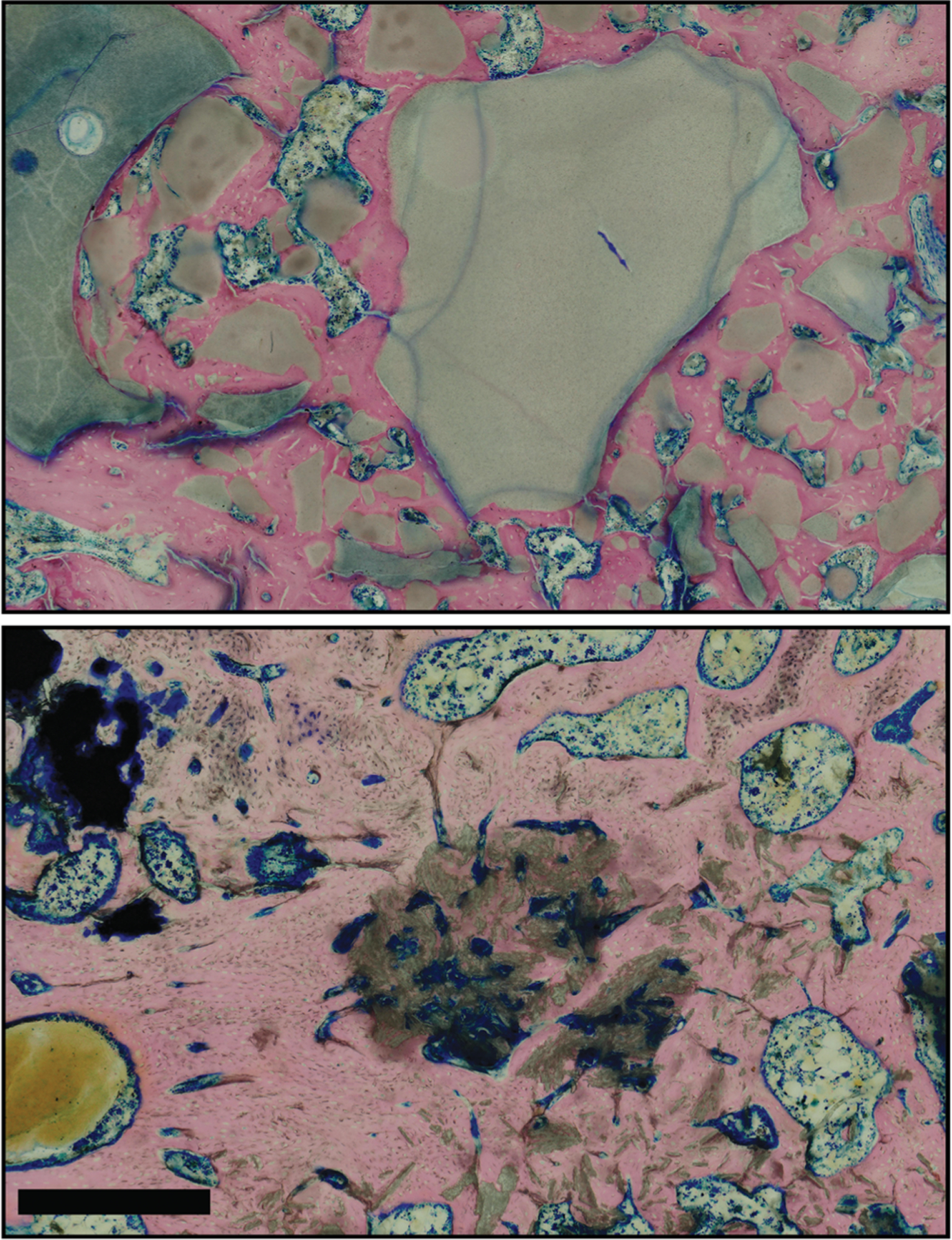
These photomicrographs show sections of the femurs stained with Sanderson’s rapid bone stain and acidified eosin. The top image shows the fluorapatite (FA) scaffold, while the bottom image shows the hydroxyapatite (HA) scaffold. The tissue surrounding the HA scaffold (gray) exhibited a higher concentration of infiltrating immune cells compared to the FA scaffold (gray) (top). The scale bar represents approximately 500 μm.

Representative sets of photomacrographs and photomicrographs for each group are shown in Figure 2. The histological images from the defect-only group confirmed the micro-CT findings, showing that, within this follow-up period of 12 weeks, a relatively small region of mineralized bone tissue was found within the defect. (Figure 2A–C). In the autograft group, the transplanted autograft tissues appeared to be incorporated into the surrounding trabecular bone structure and bridged the defects—maintaining the native tissue structure of trabecular bone with intertrabecular spaces (Figure 2D–F). All defects were filled with viable bone tissues in apatite groups (HA, FA, and FA + SVF groups, Figure 2G–O), confirming new bone tissue ongrowth and ingrowth without interposing fibrous capsules. Compared to the FA group, a greater cell density of immune cells was seen near the periphery of the HA and within the intra-scaffolding spaces (Figure 5). Interestingly, the bone deposited between the scaffold granules within the FA + SVF group (Figure 2M–O) more resembled a trabecular bone architecture, containing relatively more intra-trabecular spaces filled with fat cells and blood vessels than the FA group (Figure 2J–L).

## 4. | DISCUSSION

As stated in the introduction, this study was designed to test the overall in vivo efficacy of sintered FA scaffolds, with and without SVF, to regenerate and bridge surgically created bone defects in rat femora. Data were compared to isograft bone—similar to the current gold standard of care, autograft—and HA scaffold prepared from predicate, commercial HA powder. Micro-CT analyses and histological data collectively supported the tested hypothesis. For all three apatitegraft-treated groups, the VOIs were filled with significantly higher (<0.05) bone volumes, in terms of BVF, when compared to the defect-only and/or autograft groups. No significant differences were found between the apatite groups (Figure 4). The architecture of the newly deposited bone within the FA + SVF groups appeared to be highly similar to the surrounding trabecular bone tissue (Figure 2M–O), when contrasted to the bone formed in the defects of the FA group, the latter appearing to be denser (Figure 2J–L). Within all apatite groups, both the micro-CT and the histological images showed that the apatite-treated defects contained higher volumes of bone tissues on the surface and in between the scaffolds’ pores when compared to the surrounding trabecular space. In all samples, the defect clearly remained in the growth plate at 12 weeks. Additionally, the HA scaffold appeared to degrade faster than the FA groups, as demonstrated by the greater interdigitation of bone into the scaffold in the histological images (Figure 5).

Characterization data revealed that the in-house synthesized FA powder only had a Ca/P ratio of 1.42, which is not the theoretical stoichiometric ratio of 1.67. This observation indicated that the in-house synthesized FA powder was deficient in calcium ions. The literature suggests that both the presence of Ca vacancies, required to compensate for anionic impurities, and the incorporation of various cationic impurities (i.e., Si, Mg, Mn) during aqueous conditions may contribute to low Ca/P ratio.^31^ Interestingly, the FTIR data demonstrated the presence of some contaminants, such as carbonates, nitrates, and hydrogen phosphate. The latter, indicated by a small peak at 896 cm^−1^, has been attributed elsewhere to either hydrogen phosphate (HPO_4_^2−^) from dicalcium phosphate anhydrous (monetite, CaHPO_4_), or alternatively, to carbonate.^32^ Theoretically, a biphasic composite is anticipated for apatites synthesized with Ca/P ratio of <1.50.^32^ However, XRD analysis revealed the primary composition was of FA. It is worth noting that the highest intensity peaks associated with β-TCP, β-Ca pyrophosphate (Ca_2_P_2_O_7_), and CaHPO_4_ were not detected.^33,34^ The conditions under which apatite was synthesized could also influence its composition. Additionally, the presence of some secondary carbonates/carbonated apatites might result from synthesis under the ambient atmospheric conditions, where dissolution of CO_2_ into the reaction vessel was probable. Such byproducts were expected in literature.^32^ In future endeavors, a synthesis process conducted in an inert atmosphere, coupled with the use of highly pure water free from metallic contaminants, could enhance the purity of the fabricated FA. Fluoride electrode data indicated fluoride to be at 70% of the theoretical values, indicating the presence of some unsubstituted OH^−^ groups. This was confirmed by the presence of stretching band at 3540 cm^−1^ in the FTIR spectra, which historically has been used to prove the presence of –OH groups in HA.^32,35^ Fluoride incorporation was also supported by finding the band at 744 cm^−1^ post-sintering (S1).^18,36^ Interestingly, the literature has reported that Ca-deficient apatites better resemble natural bone tissue and thus might promote increased osteogenic activities.^37,38^ Evidently, the noted impurities did not inhibit the regenerative potential of FA in this study.

**FIGURE 6:**
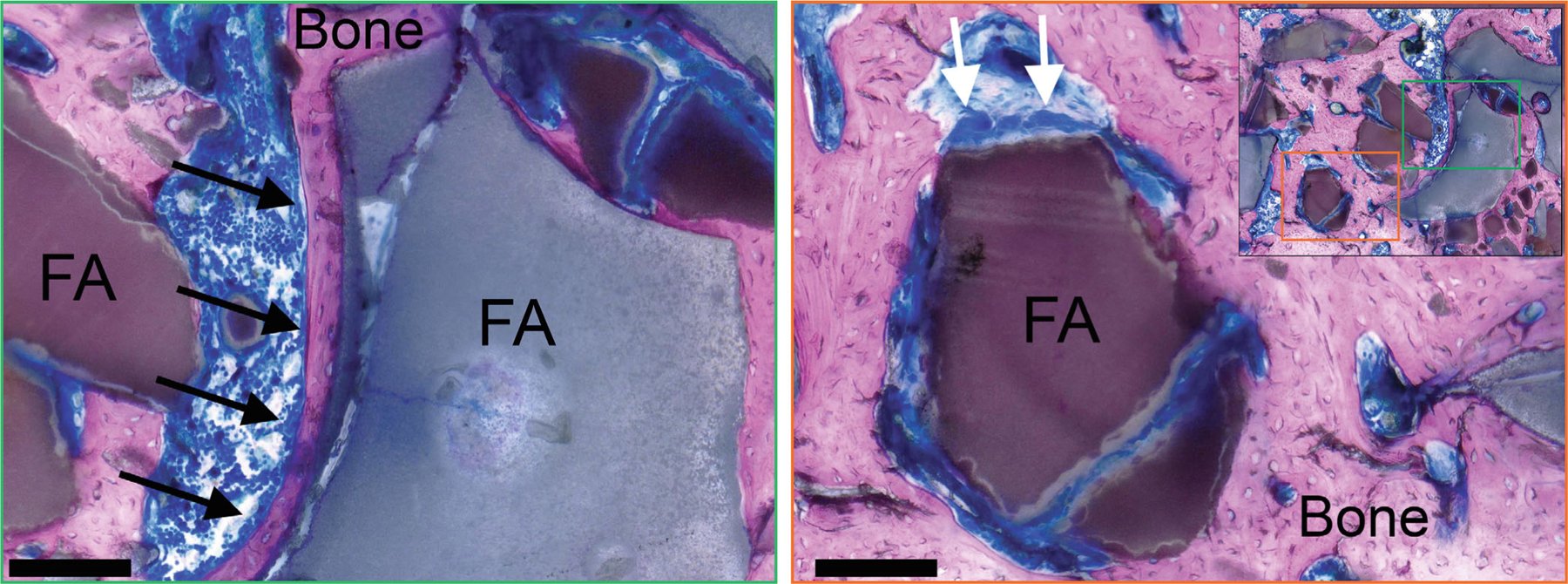
Photomicrographs of bone histological sections from the fluorapatite (FA) group stained with Sanderson’s rapid bone stain and acidified eosin. In these images, black arrows indicate what appear to be aligned osteoblasts that are attached to the FA surface (gray) or else attached to new bone deposited on the FA surfaces. White arrows indicate large, multinucleated cells attached to the FA surface, the presence of which may support the bioresorbability of FA. The scale bar represents approximately 100 μm.

The histological data revealed the absence of fibrous encapsulation of the scaffold materials and, most importantly, the presence of new bone tissue in apposition to the scaffolds for all the tested apatite (Figure 2G–O, Figures 5 and 6), confirming the osteoconductive properties of apatites. It is known that interposing fibrous tissue inhibits host bone tissue remodeling. In the literature, excessive inflammation is reported as a cause of fibrous capsulation in most engineered bio-degradable bone scaffolds, including poorly crystalline or needlelike HA,^39^ beta-TCP,^40^ and PLA/PGA biocomposites.^41^ Furthermore, histological data of the FA group revealed the attachment of large, multinucleated cells at the scaffold surface and the presence of lacunae with osteocytes within the new bone tissues, perhaps indicating the bioresorbability of the FA (Figure 6). The presence of osteocytes also suggests the deposition of healthy, maturing bone tissue capable of undergoing remodeling induced by various metabolic and mechanical cues related to the effect of the forces of daily living on bone.

Moreover, what appeared to be osteoblast-like cells were seen aligned directly on the surface of FA granules and on the surface of the bone that interfaced with the material (Figure 6). Despite the limitations associated with rodent models, the absence of bone defect healing for the defect-only group clearly demonstrated the ability of our model to differentiate between the outcomes of surgical intervention and no surgical intervention, which was supported by previous studies.^40^ This strengthens the validity of our model for assessing bone formation in defects treated with different materials, confirming that all treatment modalities used in this study had resulted in new bone deposition and remodeling.

The histological observations aligned well with what was determined by the semi-quantitative micro-CT model. Compared to HA, the FA group demonstrated slower degradation and greater bone deposition, suggesting that FA scaffolds may provide prolonged mechanical support during the bone repair process, which typically takes around 6 months to complete, an essential requirement for the clinical application of bone replacement materials in fracture care. In vitro studies have shown that fluoridation of apatites improves cell attachment, growth, and differentiation toward an osteogenic phenotype.^18,22^ Moreover, the literature indicates fluoride ion release is limited in stoichiometric FA samples that are sintered above 850°C.^18^ Despite this limitation, fluoride-releasing bone scaffolds have garnered significant attention due to their ability to improve mineralization and collagen formation in vitro and induce osteogenesis while inhibiting osteoclast maturation.^42–45^ Although the administration of free-fluoride ions has been used to improve bone mineral content, concentration control is necessary to prevent hypermineralization^46,47^ and a negative impact on the development of osteoblast cells.^48,49^ Overall, fluoride ions could stimulate bone growth in a way similar to osteogenic growth factors such as bone morphogenic proteins. This reported body of works supports the idea that fluoride-releasing materials like FA could accelerate bone deposition and may provide an explanation as to why there was a higher BVF for the FA group. Thus, one can conclude that the increased bone deposition observed in the FA group could perhaps be related to the higher rate of osteoblastic activity stimulated by the release of fluoride ions from the crystal structure. This concept requires further validation. While we feel we have determined that FA surfaces meet the Diamond Concept criteria for osteoconductivity, in future studies, it will be necessary to further characterize and evaluate the effect of fluoride release in vitro from our in-house FA scaffolds on osteogenic cells.

Apart from providing osteoconductive/osteogenic scaffolding and delivering chemical signals, potentially via fluoride ions that promote osteogenesis, an optimal bone scaffold should also offer consistent mechanical support throughout the process of bone remodeling.^50^ The premature loss of mechanical properties, resulting from issues like cracking and excessively rapid bulk degradation, poses a potential threat to effective bone healing.^51^ While we recognize the significance of mechanical assessments in evaluating biomaterials for bone repair, our scaffolds did not undergo such testing in this study. This omission was partly due to the irregular geometry of the small defects that were used in this study; the scaffolds were ultimately broken down into small granules for implantation. Future studies will incorporate thorough mechanical assessments before testing in a confirmational large animal model.

Finally, osteogenic cells are required to fulfill all Diamond concept requirements, hence the rationale for using SVF cells. Although new bone formation was similar for all apatite groups, the benefits of including SVF to enhance bone repair should not be overlooked, considering the higher bone formation seen for the FA + SVF group when compared to the isograft group. Most importantly, the SVF group also regenerated the intra-trabecular spaces with adipose cells that resembled the cancellous bone structure of the host site. The formation of intertrabecular spaces is not unexpected since SVF can undergo tri-lineage differentiation, including adipogenesis.^52^ From these data, one might conclude that the combination of SVF cells with FA scaffolds could constitute an auto-graft-like engineered bone substitute, again fulfilling the criteria defined in the Diamond Concept.

There are some limitations to this study. First, with small animal models, it is challenging to place critical-size bone defects without compromising ambulation. Also, the smaller dimension of the femur limits the accuracy of the quantitative analysis. Furthermore, the rate of bone remodeling in rats is different from that in humans.^53^ Therefore, these results will only be directly translatable to the clinics if reproducible in a relevant translational large animal model. In future studies, larger animal models will be used to test FA and HA scaffolds with identical mass, surface area, and architecture for better clinical comparison. Second, the packing density of the FA scaffoldings varied between FA and FA + SVF; the FA scaffolds were much larger than HA scaffolds. Moving forward, the scaffold volume needs to be standardized. Also, the defects were not sealed distally to prevent particle migration from the medullary canal into the knee joint, and this potential source of error needs to be resolved. Third, the resorption properties of the scaffolds need to be known and are currently being tested in an ongoing long-term study. Fourth, any cell phenotyping around the scaffolds has yet to be studied. This requires event sequences that will require new IHC methodologies. Finally, the fates of the SVF cell populations implanted within FA scaffolds remain unknown. All these limitations are currently being addressed in our ongoing studies, which include single-cell RNA sequencing.

Additionally, microCT analysis has its own limitations, although this technique provided valuable insight into identifying areas for future improvement that will enhance accuracy. The primary limitations are as follows: (1) the VOIs were smaller than the overall size of the defect due to variations in defect placement; (2) our calculations exhibited a positive bias toward the apatite groups (Figure 3) since the volumes of the scaffolding were excluded from the calculation.

The in vivo data from this study overwhelmingly support the use of heat-treated, fluoridated apatite as an engineered bone scaffold. FA scaffolds with or without SVF cells yielded equal or greater volumes of bone formation than those of clinically used apatites (HA) and autograft bone. Compared to FA alone, the inclusion of SVF cells did not increase the volume of bulk bone formed, but the regenerated tissue appeared to better resemble the native local trabecular bone architecture. With further validation in large, clinically applicable translational models, these heat-treated FA scaffolds, alone or with SVF cells, have the potential to replace autograft bone in fracture care and the repair of critical-sized bone defects. In doing so, as an off-the-shelf bone alternative to autografts, FA may eliminate the need for the additional surgical sites required to harvest autograft bone for orthopedic applications.

## Supplementary Material

S2 -Figure

S1 - Figure

## Figures and Tables

**TABLE 1:** A table showing the Ca and P contents in a known weight of fluorapatite (FA) powder as determined by inductively coupled plasma mass-spectroscopy (ICP-MS), and the concentration of fluoride as determined using a calibrated fluoride ion selective electrode (ISE) in buffered solutions of acid-dissolved FA scaffolds.

Characterization results				
	Inductively coupled plasma mass spectroscopy (ICP-MS)			Fluoride ion selective electrode
	Ca (mol/kg)	*P* (mol/kg)	Ca/P	*F* (mol/kg)
Synthesized FA	9.79	6.91	1.42	1.39
Stoichiometric FA	9.92	5.94	1.67	1.98

*Note*: The sensitivity of the ISE at measurement was ±0.098 (mol/kg) per mV.

## Data Availability

The data belongs to the Department of Veterans Affairs, available to researchers within the departmental intranet and also available upon written request to the authors.
