# Supplementary figures and images for: Sintered fluorapatite scaffolds as an autograft-like engineered bone graft
Source: J Biomed Mater Res B Appl Biomater. Author manuscript; Available in PMC 2025 Feb 14. (PMC11827050; doi:10.1002/jbm.b.35374)

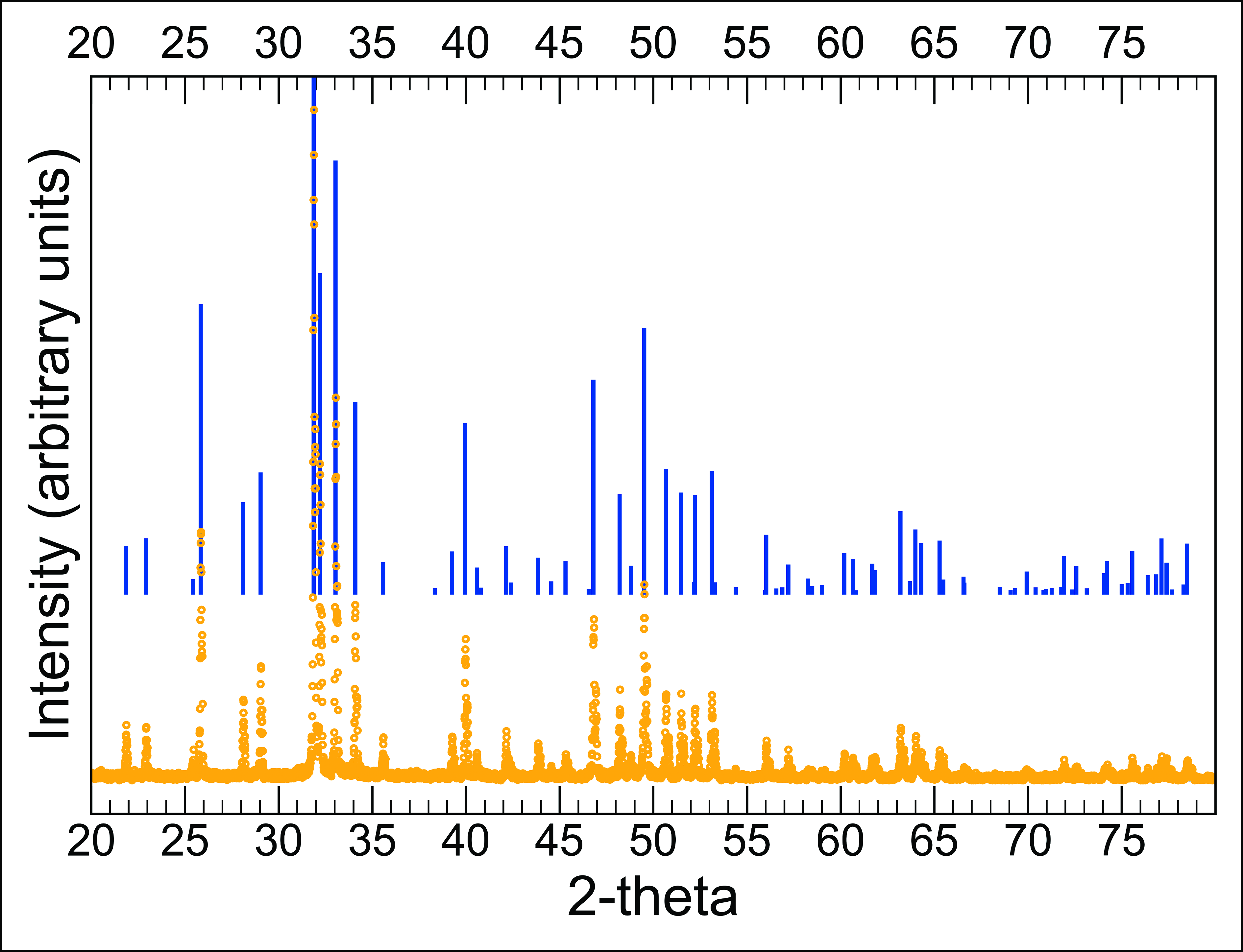

Supplement: S2 -Figure [file NIHMS2051213-supplement-S2_-Figure.tif]

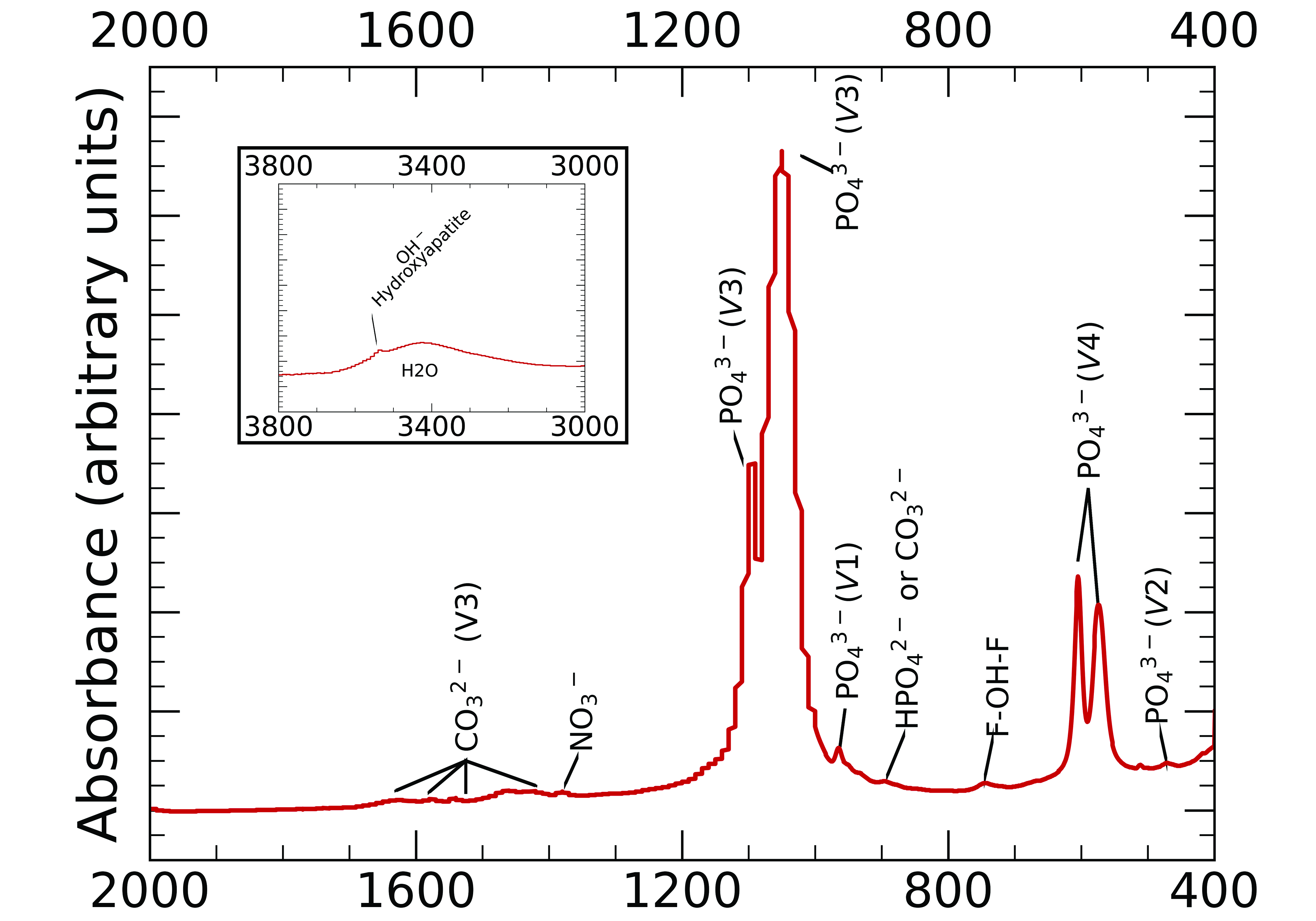

Supplement: S1 - Figure [file NIHMS2051213-supplement-S1_-_Figure.tif]
